# Dry Eye Para-Inflammation Treatment: Evaluation of a Novel Tear Substitute Containing Hyaluronic Acid and Low-Dose Hydrocortisone

**DOI:** 10.3390/biomedicines11123277

**Published:** 2023-12-11

**Authors:** Davide Borroni, Cosimo Mazzotta, Carlos Rocha-de-Lossada, José-María Sánchez-González, Antonio Ballesteros-Sanchez, María García-Lorente, Francisco Zamorano-Martín, Antonio Spinelli, Domenico Schiano-Lomoriello, Giovanni Roberto Tedesco

**Affiliations:** 1 Centro Oculistico Borroni, Gallarate, 21013 Varese, Italy; 2 Eyemetagenomics Ltd., 71–75, Shelton Street, Covent Garden, London WC2H 9JQ, UK; 3 Siena Crosslinking Center, 53035 Siena, Italy; cgmazzotta@libero.it; 4 Departmental Ophthalmology Unit, USL Toscana Sud Est l, 53100 Siena, Italy; 5 Postgraduate Ophthalmology School, University of Siena, 53100 Siena, Italy; 6 Ophthalmology Department, QVision, Vithas Almería, 04120 Almería, Spain; carlosrochadelossada5@gmail.com; 7 Ophthalmology Department, Hospital Regional Universitario Málaga, 29016 Malaga, Spain; glorentemaria@gmail.com (M.G.-L.); zamoranomartinfrancisco@gmail.com (F.Z.-M.); 8 Department of Physics of Condensed Matter, Optics Area, University of Seville, 41012 Seville, Spain; jsanchez80@us.es (J.-M.S.-G.); antbalsan@alum.us.es (A.B.-S.); 9 Department of Ophthalmology, Clinica Novovision, 30008 Murcia, Spain; 10 Sacro Cuore Ophthalmology Clinic, 87100 Cosenza, Italy; antspino@hotmail.it; 11 IRCCS Bietti Foundation, 00198 Rome, Italy; do.schiano@gmail.com; 12 Studio Oculistico Tedesco, Girifalco, 88100 Catanzaro, Italy; j.tedesco@libero.it

**Keywords:** dry eye disease, para-inflammation, hydrocortisone, corticosteroids, hyaluronic acid

## Abstract

*Purpose:* The purpose of this study was to check the efficacy and safety of a novel tear substitute containing hyaluronic acid and low-dose hydrocortisone in the treatment of moderate dry eye disease. *Methods:* In this prospective randomized study, 38 patients with moderate dry eye disease were divided into two treatment groups: Group 1 received one drop of 0.2% sodium hyaluronate and 0.001% hydrocortisone four times daily for 3 months, while Group 2 received 0.15% sodium hyaluronate and 3% trehalose at the same dosage. OSDI and SANDE questionnaires, Non-Invasive Break-Up time (NIBUT), Tear Meniscus Height (TMH), meibography, Lipid Layer Thickness (LLT), Tear Break-Up Time (TBUT), Corneal Staining Score (CFS), and Intraocular Pressure (IOP) were evaluated at baseline and after 1, 2, and 3 months of treatment. *Results:* During the treatment period, Group 1 showed statistically significant improvement in OSDI score (*p* = 0.002), SANDE score (*p* = 0.01), NIBUT (*p* < 0.0001), LLT (*p* < 0.0001), TBUT (*p* = 0.01), and CFS (*p* = 0.02). In Group 2, significant improvement was observed only in the TBUT score (*p* < 0.05). Comparison of the two groups showed that NIBUT and LLT were significantly different at the end of treatment (*p* = 0.001 for both comparisons), with more favorable results for sodium hyaluronate and hydrocortisone than for sodium hyaluronate and trehalose. No significant variations in intraocular pressure were observed in either group during the treatment period (*p* > 0.05). *Conclusions*: The study confirms that a 3-months treatment with hyaluronic acid 0.2% in combination with low-dose hydrocortisone 0.001% improves the signs and symptoms of moderate DED and that a low-dosage 0.001% hydrocortisone can be helpful in preventing the progression to chronic stages of DED.

## 1. Introduction

Dry eye disease (DED) is one of the most common ocular diseases, affecting 5–35% of the world’s population [1,2,3]. DED is considered a disorder of the ocular surface, characterized by tear film instability accompanied by possibly independent features such as clinical signs, symptoms, and visual disturbances. The ocular surface and tear film are intimately linked and loss of homeostasis of either component can trigger a self-perpetuating cycle of tear instability, tear hyperosmolarity, ocular surface inflammation, epithelial damage, and allodynia, which are the basic pathogenetic mechanisms of chronic dry eye disease [4,5]. 

In particular, inflammation plays a critical role in the pathophysiology of DED, and the expression of IL-1, TNF-α, and IL-6 is key to the inflammatory response that activates adaptive immunity and leads to chronic DED and ocular surface impairment [1,4,5,6,7,8]. Therefore, anti-inflammatory therapy, including high-dose corticosteroids, is essential for the treatment of severe DED [1,8,9,10,11,12,13,14]. However, chronic therapy with corticosteroids is associated with side effects such as increased intraocular pressure (IOP), lens opacification, and ocular surface infections [15,16,17,18,19]. 

It is noteworthy that before the vicious circle underlying DED pathogenesis is triggered, a transient para-inflammatory phase is established as a protective mechanism that can restore ocular surface homeostasis, in which cortisol seems to play a pivotal role [20,21]. 

As an endogenous glucocorticoid produced in small amounts by corneal cells, cortisol has immunomodulatory effects on the ocular surface [22,23,24]. Therefore, early treatment of mild/moderate DED with low-dose cortisol may be a valuable strategy to prevent progression from subclinical para-inflammation to overtly chronic disease [25,26].

Elucidating the pathogenesis of DED requires a multidimensional understanding of several contributing factors. The initiation of DED often starts with tear film instability, leading to tear hyperosmolarity. This hyperosmolarity, in turn, activates a cascade of inflammatory pathways involving various signaling molecules and immune cells. For instance, the activation of MAPK and NF-κB pathways leads to the upregulation of proinflammatory cytokines like IL-1, IL-6, and TNF-α. The persistent inflammation further damages the ocular epithelium and exacerbates tear film instability, creating a vicious cycle that culminates in chronic DED. Additionally, the involvement of both the innate and adaptive immune systems in DED creates a complex immunological landscape, with T-cell infiltration and activation playing a key role. Moreover, dysregulation of neural feedback mechanisms can contribute to the syndrome by impairing reflex tearing and goblet cell function, thereby worsening the condition. A deeper understanding of these mechanisms is critical for developing targeted therapies [1,5,8].

To this aim, recent studies have shown that hydrocortisone, a lipophilic analog of cortisol with low anti-inflammatory properties and short duration of action able to cross corneal barriers, effectively modulates proinflammatory cytokines on the ocular surface and improves tear film stability and production [23,24]. Furthermore, in vitro and in vivo studies have shown that low concentrations of hydrocortisone (0.001%) do not cross the corneal epithelial barrier, thus maintaining the required efficacy at the superficial level and reducing the risk of ocular side effects [23]. 

A recent randomized clinical trial investigating the pharmacokinetics of hydrocortisone confirmed these findings and demonstrated that the concentration of hydrocortisone in eye drops significantly affects penetration into the anterior chamber of the eye. Specifically, a high concentration of hydrocortisone (0.3%) results in high penetration, whereas a low concentration (0.001%) does not alter the anterior chamber concentration of cortisol, making it a safe option for longer-term treatment [27]. While several studies have explored the therapeutic effects of hyaluronic acid or hydrocortisone separately in the management of DED, the novelty of this research lies in the investigation of a unique combination of 0.2% hyaluronic acid and 0.001% hydrocortisone. This new formulation aims to synergize the moisturizing effects of hyaluronic acid with the mild anti-inflammatory properties of low-dose hydrocortisone. By doing so, our study seeks to offer a more effective and safer alternative for treating DED, targeting both tear film instability and ocular surface inflammation while minimizing potential side effects.

The aim of this study is to evaluate the efficacy of a novel tear substitute containing 0.2% hyaluronic acid and 0.001% hydrocortisone in the treatment of DED and to compare it with one of the most commonly used tear substitutes containing 0.15% sodium hyaluronate and 3% trehalose.

## 2. Materials and Methods

Thirty-eight patients with moderate dry eye disease presenting at Centro Oculistico Tedesco (Girifalco, CZ, Italy) were enrolled in this prospective randomized observational study (12 men, 26 women). The randomization of study participants was performed using a computer-generated random sequence, ensuring an unbiased allocation into the treatment and control groups. The sequence was generated by a team member not involved in patient recruitment or data analysis to maintain the integrity of the blinding process. The study protocol was approved by the local review board of the University of Messina and conformed to the tenets of the Declaration of Helsinki (approval number: 73-2022). Informed consent was obtained from all patients.

Inclusion criteria for this study consisted of the following: (I) participants must be aged over 18 years; (II) participants must have a clinically confirmed diagnosis of Dry Eye Disease (DED); (III) participants must have a Tear Break-Up Time (TBUT) of less than 10 s; and (IV) participants must have an Ocular Surface Disease Index (OSDI) score higher than 22.

Exclusion criteria were as follows: (I) individuals with a history of glaucoma; (II) individuals with allergic keratoconjunctivitis; (III) current users of contact lenses; (IV) individuals with corneal ulcers; (V) individuals who have undergone eye surgery within the past 3 months; (VI) individuals who have used topical antibiotics in the past 3 months; and (VII) individuals who have used anti-inflammatory medications, including steroids and Non-Steroidal Anti-Inflammatory Drugs (NSAIDs), within the last 3 months.

### 2.1. Treatment

Patients were divided in two treatment groups: Group 1 received 1 drop of sodium hyaluronate 0.2% (with a molecular weight of 220 kDa) and 0.001% hydrocortisone 4 times daily for 3 months, while Group 2 received 0.15% sodium hyaluronate (with a molecular weight of 220 kDa) and 3% trehalose, at the same dosage. This study utilized a single-blind approach, where the subjects were unaware of the specific products they were receiving. The bottles containing the eye drops were masked to ensure that subjects could not identify the treatment, while the physicians who evaluated the subjects were informed of the treatment details for accurate therapeutic assessment.

### 2.2. Outcomes Evaluation

At baseline and after 1, 2, and 3 months of treatment, all patients underwent the following ophthalmologic evaluations: Ocular Surface Disease Index (OSDI) questionnaire; Symptom Assessment in Dry Eye (SANDE) questionnaire; Non-Invasive Break-Up Time (NIBUT); Tear Meniscus Height (TMH); meibography; Lipid Layer Thickness (LLT); Tear Break-Up Time (TBUT); Corneal Fluorescein Staining Score (CFS) using the modified Oxford scale; and Intra Ocular Pressure measured with Corvis ST (Oculus, Wetzlar, Germany).

NIBUT, TMH, and meibography were measured using the Keratograph 5M (Oculus, Wetzlar, Germany). Specifically, NIBUT is based on the projection of Placido rings onto the corneal surface and was assessed by automatically recording the time between the last blink and the first sign of ring pattern distortion; for statistical purposes, the mean of three consecutive measurements was used. TMH was determined using the tear film scanning function of the Keratograph 5M, which allows for the capturing of images of the lower tear film meniscus and the measuring of its height. Meibography was performed on both the upper and lower eyelids to evaluate meibomian gland dropout, considering grade 0 (no gland loss), grade 1 (area of loss less than 1/3), grade 2 (area of loss between 1/3 and 2/3), and grade 3 (area of gland loss greater than 2/3), using the automatic grading function of the Keratograph 5M (JENVIS Meibo Grading Scale) [28].

LLT was measured using the LipiView II interferometer, which records a short video of the tear film interference pattern and displays data in interferometric color units (ICU), with 1 ICU representing nearly 1 nm of LLT. Maximum, minimum, and average LLT were determined from all frame averages.

TBUT was measured on a slit-lamp using a fluorescein drop prepared with a fluorescein strip, wetted with a drop of saline, and instilled onto the inferior tarsal conjunctiva. The time interval between a complete blink and the appearance of a dark spot was measured; for statistical purposes, the mean of three consecutive measurements was used. All tests using the Keratograph were conducted under controlled environmental conditions, with constant monitoring of room temperature and humidity, to mitigate the device’s known sensitivities to these variables.

### 2.3. Statistical Analysis

Although tests were performed on both eyes for each patient, only data from the right eye were included in the analysis to ensure data consistency. Numerical data are expressed as mean and standard deviation; categorical variables are expressed as absolute frequency and percentage. The fit of the data to a normal distribution was tested using the Kolmogorov–Smirnov test. The values of the variables of Group 1 and Group 2 before and after treatment were compared using the chi-square test for categorical variables, the Student’s *t*-test for parametric data, and the Mann–Whitney U test for non-parametric data. 

To evaluate differences during the treatment period, a repeated-measures analysis of variance model (ANOVA) was used that analyzed all outcome parameters. 

A *p*-value of less than 0.05 was considered statistically significant. Statistical analyses were performed using SPSS 22 software (SPSS, Inc., Chicago, IL, USA) for Windows.

## 3. Results

At baseline, no significant differences (*p* > 0.05) were observed between groups in terms of age, gender, symptoms score, and ocular surface parameters (Table 1).

### 3.1. Symptoms Evaluation

SANDE and OSDI scores decreased significantly during the treatment period in both groups (*p* < 0.05; Table 2), indicating that DED symptoms were significantly improved regardless of the type of tear substitute. The repeated-measures ANOVA only shows statistically significant differences between baseline and the 3-months group for both groups. Specifically, after 3 months of treatment, the OSDI score in Group 1 decreased by an average of 35.3 points (from 45.5 ± 28.1 points at baseline to 10.2 ± 4.3 points at 3 months of treatment), while the SANDE score in Group 1 decreased by an average of 20.9 points (from 57.1 ± 21.4 points at baseline to 36.2 ± 15.9 points at 3 months of treatment). At the same time point, Group 2 showed an average decrease in OSDI score of 17.3 points (from 44.8 ± 27.4 points at baseline to 27.5 ± 15.1 points at 3 months of treatment), while the SANDE score decreased by an average of 10.1 points (from 55.5 ± 20.3 points at baseline to 45.4 ± 17.8 points at 3 months of treatment).

Comparison of the two groups showed that OSDI scores were significantly different after 1, 2, and 3 months of treatment (*p* = 0.03, *p* = 0.03, *p* = 0.006, respectively) and that the use of sodium hyaluronate and hydrocortisone produced better results in terms of symptom reduction compared with sodium hyaluronate and trehalose.

### 3.2. Ocular Surface Assessment

**Figure 1:**
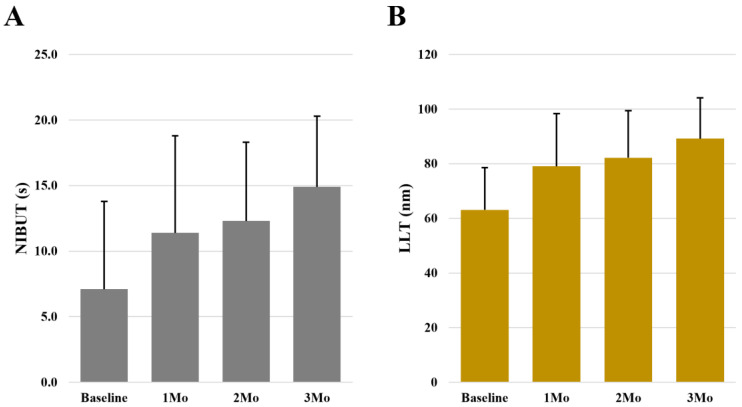
Bar plots of NIBUT (**A**) and LLT (**B**) variations during the study period in patients treated with the tear substitute containing 0.2% hyaluronic acid and 0.001% low-dose hydrocortisone (Group 1). Abbreviations: NIBUT, Non-Invasive Break-Up Time; LLT, Lipid Layer Thickness.

Of the ocular surface parameters assessed, NIBUT, LLT, TBUT, and CFS improved significantly during the treatment period in Group 1 (*p* < 0.05; Table 2). The most significant improvements were in NIBUT scores, which showed an average increase of 7.8 s after 3 months of treatment (Figure 1A), and in LLT, which improved by an average of 26.1 nm after 3 months of treatment (Figure 1B). 

In contrast, TMH and meibography scores did not show significant changes during the treatment period (*p* > 0.05; Table 2). In Group 2, significant improvement was observed only in the TBUT score (*p* < 0.05; Table 2), which showed an average increase of 0.7 s after 3 months of treatment. All other ocular surface assessment results did not show significant changes (*p* > 0.05; Table 2).

Comparison of the two groups showed that NIBUT and LLT scores were significantly different at the end of treatment (*p* = 0.001 for both comparisons), with more favorable results for sodium hyaluronate and hydrocortisone than for sodium hyaluronate and trehalose.

### 3.3. Evaluation of Safety Outcomes

No significant variations in intraocular pressure were observed in either group during the treatment period (*p* > 0.05; Table 2). In addition, no adverse events nor cataract development were reported in either group during the entire study period.

## 4. Discussion

According to DEWS II, inflammation is one of the major pathogenic mechanisms in DED^4^ and recent studies emphasize the role of para-inflammation in DED as an adaptive response of the ocular surface to restore homeostasis, which may prevent progression to more severe stages of dry eye [20,29]. When tissue dysfunction or noxious external stimuli persist over a prolonged period of time, para-inflammation can change from a protective response to a damaging process, causing a continuous inflammatory state which could eventually lead to chronic DED [20,30]. 

In this context and in agreement with the results of the present study, targeting para-inflammation may be a successful strategy for the treatment of mild/moderate DED [20]. The observed results suggest that the additional anti-inflammatory properties of low-dose hydrocortisone, combined with the moisturizing and mechanical barrier effects of hyaluronic acid, may improve the signs and symptoms of DED.

Topical treatment with pharmacological doses of hydrocortisone has previously been shown to control ocular surface inflammation by inhibiting NF-κB signaling, cytokine and chemokine production, and by regulating the expression of ICAM-1, lymphocyte apoptosis, and secretion of matrix metalloproteinases [23,24,31]. Furthermore, Bucolo et al. demonstrated that low-dose 0.001% hydrocortisone effectively modulated TNF-α, TRAIL, IL-1β, IL-8, and MMP-9 on the ocular surface both in vitro and in vivo and improved TBUT and Schirmer’s test in a rabbit model of dry eye [23].

The tear substitute tested in this study also contains hyaluronic acid 0.2%, a natural polymer whose mechanical properties and contribution to re-epithelization have been shown to improve tear film stability and tear turnover, as well as to reduce inflammatory cytokine concentrations when high-molecular-weight formulations are used [10,11,12,32]. Despite these remarkable effects, hyaluronic acid alone has already been shown to be inferior to the tear substitutes tested in improving symptoms and reducing TBUT in DED patients, suggesting a possible critical role for the low-dose hydrocortisone component in controlling subclinical inflammation and restoring ocular surface homeostasis [25].

Accordingly, we found a statistically significant improvement in tear film stability throughout the treatment period (i.e., NIBUT, LLT, TBUT, CFS) and a more significant improvement in DED symptoms (i.e., OSDI score) in patients treated with the tear substitute containing 0.2% hyaluronic acid and 0.001% hydrocortisone, compared with control patients treated with 0.15% hyaluronic acid and 3% trehalose, a treatment already known to improve DED symptoms by increasing eye lubrication. The observed improvement in treatment outcomes compared with control subjects (i.e., OSDI score, NIBUT, and LLT) suggests that lubrication alone, even when enhanced by the use of a combination of moisturizing and osmoprotective agents (i.e., hyaluronic acid and trehalose), may not be sufficient. By mimicking the physiological role of cortisol in restoring ocular surface homeostasis, the addition of low-dose hydrocortisone may be necessary to improve both symptoms and clinical outcomes.

In this study, we also found that patients treated with 0.2% hyaluronic acid and 0.001% hydrocortisone had significant improvement in LLT during the treatment period. Although the changes in meibography were not significant, it is likely that meibography provides information about the presence of anatomical alterations that, once they occur, cannot be reversed. However, the observed improvement in LLT can be taken as an indication of effective restoration of physiological conditions and improvement in meibomian gland function and meibum secretion and even of a more important role in the prevention of further loss of meibomian gland functional units on a degenerative phlogistic basis, thus favoring the transition to severe and chronic DED forms. We hypothesize that the tear substitute tested reduces meibomian gland and ocular surface inflammation, especially in patients with evaporative DED and meibomian gland dysfunction, in which bacterial lipases increase free fatty acid concentration, leading to an inflammatory response and apoptosis of epithelial cells on the ocular surface [33,34,35].

Regarding product safety, the use of low-dose hydrocortisone has been shown to be safe in a rabbit model, in which the drug was unable to penetrate the corneal epithelium and was undetectable in the aqueous humor [23]. This was also confirmed by a clinical pharmacokinetics study showing that in patients treated with low-dose hydrocortisone, its concentration remained essentially stable during the study period and was not significantly different from the endogenous cortisol levels normally found in the aqueous humor [27].

Accordingly, we also observed no significant increase in intraocular pressure after treatment, a side effect caused by increased corticosteroid concentration in the aqueous humor. Therefore, in agreement with previous clinical data demonstrating the safety of the product even over a long period of time (i.e., 6 months) [25], our results confirm that low-dose hydrocortisone can be considered safe when used as a supplementary component of the tear substitute.

A limitation of the study is the small number of participants. In the future, larger randomized controlled trials could be conducted to further confirm the conclusions of the study. In summary, our results confirm the efficacy and safety of a novel tear substitute containing 0.2% hyaluronic acid and 0.001% hydrocortisone in the treatment of moderate/severe DED over a 3-month period. Compared to conventional tear substitutes, the tested product might have the ability to restore ocular surface homeostasis by increasing cortisol levels and thereby regulating para-inflammation in addition to the conventional moisturizing effect. We acknowledge that our study is limited in that it only compares the efficacy of 0.2% sodium hyaluronate and 0.0001% hydrocortisone to 0.15% sodium hyaluronate and 3% trehalose. The inclusion of established eye drops containing anti-inflammatory agents for standard treatment of dry eye para-inflammation could provide a more comprehensive evaluation, and this is an avenue we suggest for future research.

We acknowledge that the current study did not evaluate the penetration of hydrocortisone into the aqueous humor, an important factor for confirming safety. This limitation will be considered in future research to further substantiate the safety profile of the formulation.

## 5. Conclusions

The combined effect of the restorative function of low-dose hydrocortisone and the protective function of hyaluronic acid may provide the basis for improving tear film stability and reducing ocular surface damage, ultimately preventing progression to more severe stages of DED.

## Figures and Tables

**Table 1:** Demographic and clinical characteristics of the study populations at baseline. Abbreviations: HA, hyaluronic acid; HC, hydrocortisone; TH, trehalose; OSDI, Ocular Surface Disease Index; NIBUT, Non-Invasive Break-Up Time; TMH, Tear Meniscus Height; LLT, Lipid Layer Thickness; TBUT, Tear Film Break-Up Time; CFS, Corneal Fluorescein Staining Score; IOP, Intraocular Pressure. All data are reported as mean ± standard deviation. Bold character for *p* < 0.05.

	Group 1 (HA 0.2% + HC 0.001%)	Group 2 (HA 0.15% + TH 3%)	*p*-Value
Age, years	59.6 ± 7.5	60.2 ± 8.1	0.31
Male/Female ratio	7/12	5/14	0.24
OSDI, score	45.5 ± 28.1	44.8 ± 27.4	0.72
SANDE, score	57.1 ± 21.4	55.5 ± 20.3	0.52
NIBUT, s	7.1 ± 6.7	7.7 ± 7.2	0.85
TMH, mm	0.3 ± 0.1	0.3 ± 0.1	0.74
LLT, nm	63.1 ± 15.5	64.2 ± 14.8	0.39
TBUT, s	3.2 ± 1.1	3.4 ± 1.4	0.61
CFS, score	2.2 ± 0.7	2.1 ± 0.8	0.59
Meibography, score	1.6 ± 0.4	1.7 ± 0.5	0.41
IOP, mmHg	16.1 ± 2.6	16.5 ± 2.9	0.21

**Table 2:** Symptoms Scores and Ocular Surface Parameters Evaluated at Baseline and After Treatment. Abbreviations: HA, hyaluronic acid; HC, hydrocortisone; TH, trehalose; OSDI, Ocular Surface Disease Index; NIBUT, Non-Invasive Break-Up Time; TMH, Tear Meniscus Height; LLT, Lipid Layer Thickness; TBUT, Tear Film Break-Up Time; CFS, Corneal Fluorescein Staining Score; IOP, Intraocular Pressure. All data are reported as mean ± standard deviation. Bold character for *p* < 0.05.

Group 1 (HA 0.2% + HC 0.001%)					
	Baseline	1Mo	2Mo	3Mo	*p*-Value
OSDI, score	45.5 ± 28.1	16.7 ± 7.1	13.3 ± 5.9	10.2 ± 4.3	**0.002**
SANDE, mm	57.1 ± 21.4	42.1 ± 18.9	40.4 ± 16.8	36.2 ± 15.9	**0.01**
NIBUT, s	7.1 ± 6.7	11.4 ± 7.4	12.3 ± 6	14.9 ± 5.4	**<0.0001**
TMH, mm	0.3 ± 0.1	0.3 ± 0.1	0.27 ± 0.2	0.28 ± 0.14	0.5
LLT, nm	63.1 ± 15.5	79.1 ± 19.3	82.2 ± 17.2	89.2 ± 14.9	**<0.0001**
TBUT, s	3.2 ± 1.1	3.9 ± 1.3	4.3 ± 1.2	4.9 ± 1.4	**0.01**
CFS, score	2.2 ± 0.7	2.1 ± 0.8	2.0 ± 0.8	1.8 ± 0.7	**0.02**
Meibography, score	1.6 ± 0.4	1.6 ± 0.4	1.6 ± 0.4	1.6 ± 0.5	0.58
IOP, mmHg	16.1 ± 2.6	16 ± 2.5	15.8 ± 2.6	15.7 ± 2.7	0.38
**Group 2 (HA 0.15% + TH 3%)**					
	**Baseline**	**1Mo**	**2Mo**	**3Mo**	***p*-value**
OSDI, score	44.8 ± 27.4	32.7 ± 15.2	33.2 ± 15.9	27.5 ± 15.1	**0.01**
SANDE, mm	55.5 ± 20.3	50.1 ± 19.6	48.4 ± 18.3	45.4 ± 17.8	**0.03**
NIBUT, s	7.7 ± 7.2	9.3 ± 8.8	11.4 ± 5.8	11.5 ± 5.4	0.08
TMH, mm	0.3 ± 0.1	0.3 ± 0.1	0.29 ± 0.1	0.28 ± 0.14	0.62
LLT, nm	64.2 ± 14.8	64.7 ± 16.2	70.4 ± 18.9	69.5 ± 16.9	0.15
TBUT, s	3.4 ± 1.4	3.6 ± 1.3	3.9 ± 1.5	4.1 ± 1.5	**0.04**
CFS, score	2.1 ± 0.8	2.1 ± 0.7	2.1 ± 0.8	2.0 ± 0.8	0.12
Meibography, score	1.7 ± 0.5	1.7 ± 0.4	1.7 ± 0.5	1.6 ± 0.5	0.64
IOP, mmHg	16.5 ± 2.9	16.2 ± 2.8	16.6 ± 2.8	15.9 ± 2.3	0.31

## Data Availability

The data presented in this study are available on request from the corresponding author.
